# Dietary Nitrate Ingestion Does Not Improve Neuromuscular Performance in Male Sport Climbers

**DOI:** 10.5114/jhk/161812

**Published:** 2023-04-20

**Authors:** Luis A. Berlanga, Alvaro Lopez-Samanes, Julio Martin-Lopez, Ruben Martinez de la Cruz, Marta Garces-Rimon, Justin Roberts, Gabriele Bertotti

**Affiliations:** 1 Physical Activity and Sport, Centro de Estudios Universitarios Cardenal Spínola CEU, Sevilla, Spain.; 2 Exercise Physiology Group, Faculty of Health Sciences, Universidad Francisco de Vitoria, Madrid, Spain.; 3 Physiotherapy, Sputnik Climbing, Madrid, Spain.; 4 Grupo de Investigación en Biotecnología Alimentaria, Universidad Francisco de Vitoria, Madrid, Spain.; 5 Cambridge Centre for Sport and Exercise Sciences (CCSES), School of Psychology and Sport Science, Anglia Ruskin University, East Road, Cambridge, UK.

**Keywords:** nitric oxide, climbing, sport performance, dietary supplements

## Abstract

Beetroot juice (BJ) is commonly used as an ergogenic aid in endurance and team sports, however, the effect of this supplement on climbing performance is barely studied. The purpose of the current study was to investigate the effect of acute BJ ingestion on neuromuscular and biochemical variables in amateur male sport climbers. Ten physically active sport climbers (28.8 ± 3.7 years) underwent a battery of neuromuscular tests consisting of the half crimp test, the pull-up to failure test, the isometric handgrip strength test, the countermovement jump (CMJ) and the squat jump (SJ). Participants performed the neuromuscular test battery twice in a cross-over design separated by 10 days, 150 min after having consumed either 70-mL of BJ (6.4 mmol NO_3-_) or a 70-mL placebo (0.0034 mmol NO_3-_). In addition, nitrate (NO_3-_) and nitrite (NO_2-_) saliva concentrations were analysed, and a side effect questionnaire related to ingestion was administrated. No differences were reported in particular neuromuscular variables measured such as the CMJ (p = 0.960; ES = 0.03), the SJ (p = 0.581; ES = −0.25), isometric handgrip strength (dominant/non dominant) (p = 0.459–0.447; ES = 0.34–0.35), the pull-up failure test (p = 0.272; ES = 0.51) or the maximal isometric half crimp test (p = 0.521–0.824; ES = 0.10–0.28). Salivary NO_3-_ and NO_2-_ increased significantly post BJ supplementation compared to the placebo (p < 0.001), while no side effects associated to ingestion were reported (p = 0.330–1.000) between conditions (BJ/placebo ingestion). Acute dietary nitrate supplementation (70-mL) did not produce any statistically significant improvement in neuromuscular performance or side effects in amateur sport climbers.

## Introduction

Rock climbing is the branch of climbing in which participants climb up, down or across natural rock formations or non-natural rock walls (Sheel, 2004). In the last decades, indoor rock-climbing denominated sport climbing has increased in popularity in developed countries and has been introduced as a contemporary discipline (Sas-Nowosielski, 2021) in the Tokyo 2020 Olympic Games programme (MacKenzie et al., 2020). According to the International Federation of Sport Climbing (IFSC), 25 million of people climb regularly worldwide and the number is growing significantly every year (Saul et al., 2019). Thus, sports climbing has attracted the attention of researchers and sports scientists in terms of understanding the determinants of physical performance. Physical determinants of performance in rock climbing have specifically been related to high values of upper-limb strength (e.g., finger and handgrip strength) (Laffaye et al., 2016; MacKenzie et al., 2020). Moreover, Levernier et al. (2021) established that the rate of force development (RFD) is one of the principal neuromuscular determinants in rock climbing due to the reduced time to grip strongly during movements, especially measured at 200 ms during the maximal voluntary isometric contraction during the half (i.e., the angle of the proximal interphalangeal is 90° with an extension for the distal interphalangeal) or the slope crimp test (i.e., flexion of the distal interphalangeal and a little flexion of the proximal interphalangeal) (Levernier and Laffaye, 2021). Thus, due to high neuromuscular demands which characterize high climbing performance, the identification of dietary supplements that may enhance neuromuscular performance could be a valuable strategy to be adopted by climbers.

Beetroot juice (BJ) provides a rich source of inorganic nitrate (NO_3-_), which is why its consumption is increasing in popularity as a sports dietary supplement due to its effectiveness in improving endurance (Gao et al., 2021) and neuromuscular performance (i.e., concentric/isometric/eccentric muscle power performance) (Coggan et al., 2021; San Juan et al., 2020). NO_3-_ contained in BJ can be reduced to nitrite (NO_2-_) by anaerobic bacteria in the oral cavity and once NO_2-_ is swallowed, it is decomposed to nitric oxide (NO) in the stomach (Jones et al., 2021). The resultant NO is a potent vasodilator compound that facilitates blood flow in skeletal muscle enhancing muscle oxygenation (Joyner and Dietz, 1997), increasing intramuscular nitrate storage (Nyakayiru et al., 2020) improving contractile force in type II (fast-twitch) muscle fibres (Jones et al., 2016) or even emerging evidence suggesting that dietary NO_3-_ may enhance cognition and brain perfusion (Presley et al., 2011). These mechanisms could explain a possible increment in neuromuscular performance previously reported (Jurado-Castro et al., 2022; Rodriguez-Fernandez et al., 2021). However, evidence in high-intensity efforts is controversial with previous research reporting performance improvements (Wylie et al., 2013), in contrast with other authors who did not observe any neuromuscular performance improvements in short high-intensity efforts using BJ supplement versus a placebo (Jonvik et al., 2021). These discrepancies between studies may be due to different aspects such as BJ doses used, the sports level of participants (amateur vs. professional), the ingestion mode (acute vs. chronic) or the type of efforts measured (i.e., time-trial vs. all-out test) (Senefeld et al., 2020).

Although some dietary supplements have been found useful for improving rock climbing performance such as New Zealand blackcurrant (Fryer et al., 2020), beta-alanine (Sas-Nowosielski et al., 2021; Sas-Nowosielski and Kaczka, 2022), creatine (Doran and Godfrey, 2001; Szczęsna-Kaczmarek, 2016) and beta-alanine/sodium citrate in combination (Sas-Nowosielski and Kaczka, 2022), to our knowledge, no previous studies have been undertaken to study the effects of BJ supplementation on neuromuscular performance in rock climbers. Thus, the aim of this double-blind crossover study was to determine whether acute supplementation of 70-mL concentrated BJ (6.4 mmol of NO_3-_) 150-min before a specific neuromuscular test battery would improve sport climbing performance. We hypothesized that this single dose of BJ would be effective to enhance neuromuscular performance in the half crimp or the pull-up to exhaustion test.

## Methods

### Participants

Ten amateur male sport climbers (age: 22.8 ± 5.1 years; body mass: 67.0 ± 5.6 kg; body height: 1.7 ± 0.1 m; body mass index: 25.1 ± 1.7; sport climbing experience: 2.8 ± 0.7 years; climbing level: ≥ 6c) between 18 and 40 years old were eligible for participating in this study. Exclusion criteria were suffering an injury in the three months prior to the investigation, training experience below 2 years, the use of medicines or dietary supplements, or intolerance to BJ. After being fully informed of the experimental protocols, all participants provided their informed written consent to participate. The study procedures were approved by the Human Ethics Committee of the Francisco de Vitoria University (protocol code 15/2021, approved on September 2021) and complied with the Declaration of Helsinki.

### Experimental Design

**Figure 1:**
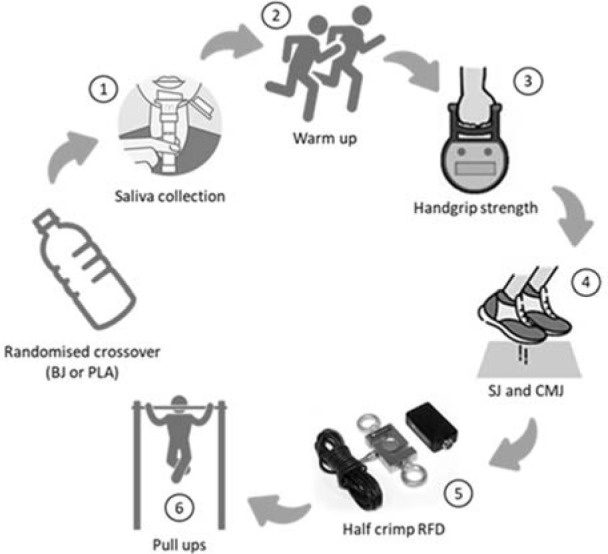
. Experimental design.

An *a priori* sample size calculation using G*Power (version 3.1.9.2; University of Düsseldorf; Düsseldorf, Germany) indicated that 8 participants were needed with an effect size of 0.8, α of 0.05, and power of 0.8 to determine differences between two dependent means according to previous research (Clifford et al., 2016). A randomized, double-blind, placebo-controlled crossover experimental design was employed (in each trial, 50% of participants ingested BJ and 50% ingested a placebo) with random assignment to each supplement (Research Randomizer, www.randomizer.org, accessed on the 4^th^ of October, 2021) as reported in previous studies utilizing BJ supplementation (Lopez-Samanes et al., 2020), and the study was registered and released in ClinicalTrials.gov (identifier NCT05210244). One hundred and fifty minutes after the intake of BJ or the placebo, a saliva sample for determining NO_3-_ and NO_2-_ levels was obtained, and afterwards male rock climbers underwent two same testing sessions separated by ten days, to allow enough time for beetroot juice or the placebo wash-out. Participants were allocated to receive a 70-mL dose of BJ containing 6.4 mmol of NO_3-_ (Beet-It-Pro Elite Shot, James White Drinks Ltd., Ipswich, UK) or 70-mL nitrate-depleted BJ placebo (0.0034 mmol of NO_3-_), matched in flavour, appearance, and packaging (Beet-It-Pro Elite Shot, James White Drinks Ltd., Ipswich, UK). Prior to the assessment, participants were requested to attend a preliminary assessment of body composition and underwent a familiarization session during the warm-up (Levernier and Laffaye, 2019). Testing sessions included a neuromuscular test battery consisting of the countermovement and the squat jump, the isometric handgrip strength test, the pull-up failure test and the half crimp test (Figure 1). Experimental procedures were performed at the same time of the day to minimise any influence of circadian rhythms as previously reported in other studies on intermittent sports performance (Martin-Lopez et al., 2022) or BJ time-of-day ingestion (Dumar et al., 2021). Environmental conditions were similar between trials (21.0 ± 0.5°C, 38–40% humidity), measured using a portable weather station (Metereological Station, Kunken, Spain). Thirty minutes after the completion of the neuromuscular test battery, a rating of perception effort scale (RPE, 0–10 points) was administrated to establish the perceived internal load for each testing session (Foster et al., 2001).

### Nutritional Intervention

All participants were instructed to follow a diet sheet (with a specific recommendation regarding the carbohydrate, fat and protein intake) 24 hours before each testing day and limiting food sources with high NO_3-_ concentrations (e.g., beetroot, celery, or spinach) 48 hours before each testing day based on previous recommendations (Dominguez et al., 2017). In addition, 24 hours prior to each session, participants were encouraged to avoid brushing their teeth, using an oral antiseptic rinse or ingesting gum, sweets or stimulants (e.g., caffeine) that could affect the oral microbiota and interfere with NO_3-_ reduction (Bescos et al., 2020). In addition, saliva samples were obtained for determining NO_3-_ and NO_2-_ saliva concentrations after BJ/placebo consumption. After the completion of the neuromuscular test battery, in the following morning, participants were provided with a survey to be filled out regarding side effects induced by BJ or the placebo ingestion (e.g. nausea, red urine or reflux) based on previous research (Wickham et al., 2019).

### Maximal Isometric Half-Crimp Test

Rock climbers stood motionless, one hand positioned on the portable strain gauge (Chronojump Boscosystems, Barcelona, Spain) sampling at 80 Hz (Buendia-Romero et al., 2021) and the opposite arm hanging alongside the body. The angle between the arm and the chest was 90° in the sagittal plane, and the angle between the arm and the forearm was set at 90° (Levernier and Laffaye, 2021). A visual control of the angle before the climber began their action on the dynamometer was used to check the postural position. Height of the measurement’s apparatus was set according to each climber, with participants then performing a maximal finger flexion on a dynamometer in a half crimp position (i.e., the angle of the little flexion of the proximal interphalangeal was 90° with an extension for the distal interphalangeal) (Levernier and Laffaye, 2019) on the 20 mm rung. In addition, participants applied a vertical force directly on the dynamometer with a surface of the contact between the fingers and the dynamometer, and were instructed to apply vertical force downwards on the dynamometer with their fingers as tightly and as quickly as possible with each hand twice. RFD values at 50 ms, 100 ms, 200 ms and at 95% of maximal force during the half crimp were recorded. A 3-min rest interval was allowed between each trial, and maximum values obtained were recorded for subsequent statistical analysis.

### Pull-Up Failure Test

The pull-up failure test was performed with a prone grip and hands were separated by a distance equivalent to the participant’s acromion-to-acromion length. In order to consider a repetition valid, the participant needed to start the movement hanging on the bar with the elbows fully extended and the feet in the air with the knees flexed and the hip in a neutral position (Munoz-Lopez et al., 2017). After holding that position for 2 s, participants were encouraged to perform the pull-up as fast as possible until their chins were above the bar until failure. Only properly completed repetitions were considered for subsequent analysis.

### Isometric Handgrip Strength

Maximal isometric handgrip strength was measured for the dominant and non-dominant hands using a calibrated handgrip dynamometer (Takei 5101, Tokyo, Japan). Volunteers were in an upright position, with the tested arm in front of the body in an extended position, the contralateral arm beside the body, and the forearm and the hand in a neutral position (Fernandez-Elias et al., 2022). The highest value obtained was used for further analysis.

### Countermovement and Squat Jumps

The countermovement jump (CMJ) was performed using a commercially available jump mat (Chronojump Boscosystems, Barcelona, Spain) previously validated (Pueo et al., 2020). The CMJ was initiated from a stationary standing position, followed by a 90º knee flexion and the jump phase. Rock climbers were asked to keep their hands on hips during the entire CMJ and perform two attempts with a 1-min rest interval between trials (Lopez-Samanes et al., 2018). The squat jump (SJ) started with the trunk straight and knee flexion at 90º, feet and shoulder width apart and maintaining the position for at least 2 s. This was followed by the participant completing a vertical jump as high as possible with legs extended, performing two attempts with a 1 min rest interval between trials (Petrigna et al., 2019). The mean value of the two attempts was recorded as the maximum jump and used for subsequent statistical analysis, unless the consecutive measures differed by more than 5%. If necessary, additional jump attempts were performed until two consecutive measures differed less than 5%.

### Saliva NO_3-_ and NO_2-_ Measurements

Saliva samples were collected 150 min after BJ or the placebo ingestion and were stored at −20°C for four months until subsequent analysis. To confirm the effectiveness of BJ supplementation on nitrate (NO_3-_) and nitrite (NO_2-_) levels, saliva concentrations were measured using a nitric oxide assay kit (EMSNO K195325, Thermo Fisher Scientific, Roskilde, Denmark) according to the manufacturer’s instructions and as reported in previous literature (Richard et al., 2018). All samples and standards were measured in duplicate and averaged.

### Statistical Analysis

The Shapiro-Wilk test confirmed the normal distribution of all data and each variable was presented as mean ± SD. All variables were compared between BJ and placebo testing sessions using paired-sample *t*-tests with statistical significance set at *p* ≤ 0.05. Cohen’s *d* effect sizes (ES) (± 95% confidence intervals [CI]) were also determined to quantify the magnitude of differences between sessions for each variable and interpreted based on the following criteria: trivial = 0–0.19, small = 0.20–0.49, medium = 0.50–0.79, and large ≥ 0.80; with ES calculated using an established Microsoft Excel spreadsheet (Lakens, 2013). The McNemar’s test was also used to detect differences in the prevalence of side effects. Calculations and figures were made using Graph Prism software (version 8.0.1, GraphPad Software, Inc., San Diego, CA).

## Results

### Maximal Isometric Half Crimp Test

In comparison to the placebo, no differences were reported in the isometric half crimp test in the dominant hand in the RFD values at 50 ms (1821.37 vs. 1701.59 N·s^-1^; 10.3 ± 23.0%; *p* = 0.534; ES = 0.27 [0.00; 0.55]), 100 ms (1359.97 vs. 1289.50 N·s^-1^; 7.5 ± 14.4%; *p* = 0.576; ES = 0.28 [0.00; 0.56]), 200 ms (703.2 vs. 678.3 N·s^-1^; 5.5 ± 18.6%; *p* = 0.647; ES = 0.21 [−0.07; 0.48]), and 95% of maximal force during the half crimp test (116.7 vs. 127.7 N·s^-1^; 14.8 ± 18.6%; *p* = 0.556; ES = 0.26 [−0.02; 0.54]). No differences between conditions were reported neither in the isometric half crimp test in the non-dominant hand in the RFD values at 50 ms (1538.1 vs. 1639.9 N·s^-1^; 4.5 ± 16.7%; *p* = 0.521; ES = −0.28 [−0.56; −0.01]), 100 ms (1150.96 vs. 1183.14 N·s^-1^; 1.41 ± 13.9%; *p* = 0.726; ES = −0.16 [−0.43; −0.12]), 200 ms (655.33 vs. 666.40 N·s^-1^; *p* = 0.824; ES = 0.10 [−0.18; −0.37]), and 95% of maximal force (106.9 vs. 104.4 N·s^-1^; 3.6 ± 12.5%; *p* = 0.859; ES = 0.08 [−0.20; 0.36]).

### Pull-Up Failure Test

**Figure 2:**
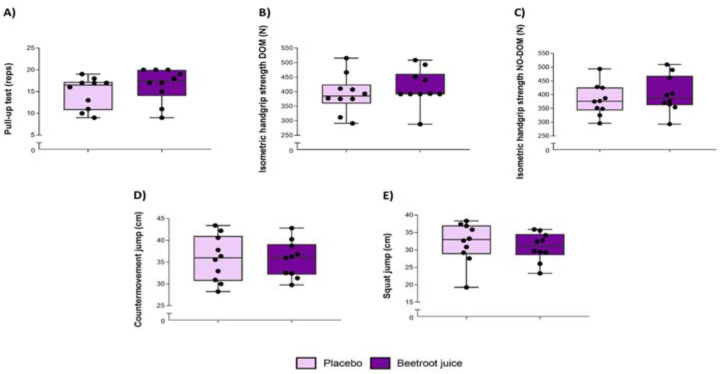
. Neuromuscular test battery with the ingestion of 70 mL of beetroot juice (6.4 mmol NO_3-_) or a placebo (0.0034 mmol NO_3-_) in male rock climbers.

No significant differences were obtained between BJ and placebo conditions in the pull-up until failure test (+13.6 ± 12.2%; *p* = 0.272; ES = 0.51 [0.23; 0.79]) (Figure 2A).

### Isometric Handgrip Strength

No statistical differences were reported in the isometric handgrip strength test for the BJ vs. the placebo condition in the dominant (+7.2 ± 20.4%; *p* = 0.459; ES = 0.34 [0.06; 0.62]) (Figure 2B) and the non-dominant hand (+5.8 ± 10.3%; *p* = 0.447; ES = 0.35 [0.07; 0.63]) (Figure 2C).

### Countermovement and Squat Jumps

In comparison to the placebo, no statistical differences were reported for acute BJ ingestion in CMJ height (+0.3 ± 7.7%; *p* = 0.960; ES = 0.03 [−0.31; 0.25]) (Figure 2D) or the SJ (−2.2 ± 14.2%; *p* = 0.581; ES = −0.25 [−0.53; 0.03]) (Figure 2E).

### Salivary NO_3-_/NO_2-_ Concentrations, Side Effects Questionnaire and Rate of Perception Effort

**Figure 3:**
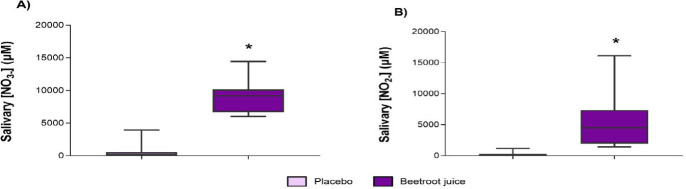
. Salivary NO_3-_ and NO_2-_ concentrations. * p ≤ 0.001

Significant differences were reported for salivary NO_3-_ (593.4 vs. 8990.5 μM; *p* ≤ 0.001; ES = 4.15 [3.65; 4.63]) (Figure 3A) and NO_2-_ (219.9 vs. 5406.9 μM; *p* = 0.001; ES = 1.65 [1.31; 1.97]) (Figure 3B) concentrations between the placebo and BJ ingestion. Only 30% of the participants (3/10 participants) correctly identified the supplement that they had received (BJ or placebo), and there were limited side effects reported apart from few cases of increased fatigue or increased urine production (*p* = 0.330–1.000) (López Samanes at al., 2022). During the hours after completion of the neuromuscular test battery, rock climbers showed a similar prevalence of side effects in the two experimental protocols (BJ vs. placebo). In addition, no differences for RPE values were reported between BJ and placebo conditions (5.7 ± 0.8 vs. 5.5 ± 0.9; *p* = 0.531; ES = 0.23 [−0.04; 0.51]).

## Discussion

The aim of this study was to investigate the acute effects of BJ intake on neuromuscular and biochemical variables in amateur male sport climbers. According to our results, in comparison to the placebo intake, acute intake of 70-mL of BJ did not show any neuromuscular improvement in male rock climbers jump height (−2.2–+0.3%), isometric handgrip strength (+5.8–+7.2%), the pull-up test until failure (+13.6 %) and the maximal isometric half crimp test (+1.7–+10.3 %). Overall, these outcomes suggest that BJ supplementation with a dose of 70-mL (6.4 mmol NO_3-_) is not an effective acute ergogenic protocol to obtain meaningful improvements in several aspects of rock-climbing performance such as isometric handgrip strength or the maximal isometric half crimp test.

Sport climbing performance is characterized by higher values of upper and lower body strength (Li et al., 2018; Michailov et al., 2018; Watts et al., 1993). Thus, the implementation of different standardized tests such as a pull-up test or the CMJ/SJ should provide a useful tool for detecting if the use of BJ could improve overall neuromuscular strength performance. Although no previous studies have analysed the acute effects of BJ (70-mL, 6.4 mmol NO_3-_) in male rock climbers, our data are in agreement with previously reported research investigating male athletes in sports disciplines with greater lower-body strength requirements (e.g., basketball or tennis) (Lopez-Samanes et al., 2020a, 2020b), that reported no enhancement in neuromuscular performance with acute BJ ingestion.

Isometric handgrip strength has been defined a secondary determinant of rock-climbing performance (MacKenzie et al., 2020), due to its importance during sport climbing characterized by continuous isometric holds to maintain and change positions (Saul et al., 2019). According to our data (+5.8–+7.2%), no benefits were observed between BJ vs. the placebo ingestion in isometric handgrip strength (i.e., dominant/non-dominant side). Our data are in agreement with previous studies which did not report changes in isometric strength in well-trained athletes (Fernandez-Elias et al., 2022) or in healthy adults (Siervo et al., 2016) after BJ consumption.

Previous studies have also determined a close relationship between the selected aspects of climbing performance and maximum pull-up capacity (MacKenzie et al., 2020). Thus, it is crucial to recognize potential ergogenic aids or dietary supplements that could improve neuromuscular performance with regard to this neuromuscular aspect. According to our data, BJ intake did not reach statistical difference in the number of pull-ups (+13.6%) compared to the placebo, although presented moderate side effects (d = 0.51) were observed, which indicates that dietary nitrate may play a role in all-out efforts such as sports climbing performance, that is in agreement with previous literature (Senefeld et al., 2020) and needs to be corroborated. Despite the fact that no previous studies focused on the effects of BJ ingestion on sport climbing performance have been developed, the ingestion of other ergogenic aids such as caffeine has demonstrated greater effects on performance compared with BJ in the pull-up test (Cabañes et al., 2013). Thus, the use of other ergogenic aids such as caffeine may provide an adjunct strategy when combined with BJ for enhancing maximum pull-up performance in rock climbers.

The ability to develop a high level of force in a short time (i.e., RFD) is also an important determinant for rock climbing performance (Levernier and Laffaye, 2021). However, only few studies have investigated this variable, despite its apparent importance (Fanchini et al., 2013; Levernier and Laffaye, 2019). According to our data, no significant differences were obtained between conditions (BJ vs. placebo) in the RFD at 50 ms, 100 ms, 200 ms and 95% of maximal force during the half crimp test (dominant/non-dominant side). In addition, no differences were reported in the RPE between conditions (5.7 vs. 5.5 points), which is in agreement with recent research where lower doses of BJ were administrated (70-mL) acutely (Fernandez-Elias et al., 2022) compared with higher doses (140-mL) (Wickham et al., 2019). Finally, no differences were detected in side effects (i.e., gastrointestinal upset) from acute BJ consumption, which differs from previous studies (Wickham et al., 2019). However, the different doses used (140-mL vs. 70-mL) could, in part, explain these results.

The current investigation has several limitations that should be discussed to enhance its applicability to real sports context scenarios. Firstly, although we selected a dose of 6.4 mmol of NO_3-_ in the experimental trial with concentrated BJ (which is above the threshold suggested to obtain ergogenic benefits above ~5 mmol NO_3-_ (Jones et al., 2021; Senefeld et al., 2020), future investigations should be undertaken with higher doses or chronic supplementation to assess the acute impact of BJ. Second, the lack of performance-enhancing benefits of BJ supplementation may be affected by the limited sample size in our study. Third, we only selected male rock climbers, thus, future studies should analyse the effect of BJ consumption on female rock climbers. Fourth, although dietary nitrate intake has been associated with faster phosphocreatine resynthesis which could delay its depletion during repetitive exercise efforts (Dominguez et al., 2018), future studies should be developed in this Olympic modality for corroborating the study findings. Fifth, while some of the tests conducted in the current study are commonly used, they may lack a degree of specificity (i.e., CMJ/SJ) or be isolated in their application (e.g., the half crimp test). Therefore, future research should consider more specific tests pertinent to sport climbing disciplines.

## Conclusions

Acute ingestion of a commercialized BJ shot (70-mL containing 6.4 mmol of NO_3-_) was ineffective in improving neuromuscular performance in amateur sport climbers. Future investigations are required to corroborate these findings, and to explore whether higher doses or chronic intake of BJ may enhance sport climbing performance.
